# Finite Element Simulation of Clubfoot Correction: A Feasibility Study Toward Patient-Specific Casting

**DOI:** 10.3390/children12101307

**Published:** 2025-09-28

**Authors:** Ayush Nankani, Sean Tabaie, Matthew Oetgen, Kevin Cleary, Reza Monfaredi

**Affiliations:** 1 Sheikh Zayed Institute, Children’s National Hospital, Washington, DC 20010, USA; 2 Nationwide Children’s Hospital, 700 Childrens Drive, Columbus, OH 43205, USA; 3 Department of Orthopedics, Children’s National Hospital, Washington, DC 20010, USA; 4 A. James Clark School of Engineering, University of Maryland, College Park, MD 20740, USA

**Keywords:** clubfoot, Ponseti method, finite element analysis (FEA), pediatric orthopedics

## Abstract

**Background:** Congenital talipes equinovarus (clubfoot) affects 1–2 per 1000 newborns worldwide. The Ponseti method, based on staged manipulations and casting, is the gold standard for correction. However, the biomechanical processes underlying these corrections remain poorly understood, as infants rarely undergo imaging. Computational modeling may offer a non-invasive approach to studying correction pathways and exploring novel applications, such as customized casts. **Methods:** We developed a proof-of-concept framework using iterative finite element analysis (iFEA) to approximate the surface-level geometric corrections targeted in Ponseti treatment. A 3D surface model of a training clubfoot foot was scanned, meshed, and deformed stepwise under applied computational loads. The model was assumed to be homogeneous and hyperelastic, and correction was quantified using Cavus, Adductus, Varus, Equinus, and Derotation angles. We also introduced a secondary adult leg 3D surface model to assess whether model simplification influences correction outcomes, by comparing a homogeneous soft tissue model with a non-homogeneous model incorporating bone structure. **Results:** In the training model, iFEA generated progressive deformations consistent with Ponseti correction, with mean angular deviations of ±3.2°. In the adult leg model, homogeneous and non-homogeneous versions produced comparable correction geometries, differing by <2° in outcomes. The homogeneous model required less computation, supporting its use for feasibility testing. Applied loads were computational drivers, not physiological forces. **Conclusions:** This feasibility study shows that iFEA can reproduce surface-level geometric changes consistent with Ponseti correction, independent of model homogeneity. While not replicating clinical biomechanics, this framework lays the groundwork for future work that incorporates clinician-applied forces, pediatric tissue properties, and patient-specific geometries, with potential applications in customized 3D-printed casts.

## 1. Introduction

**Figure 1:**
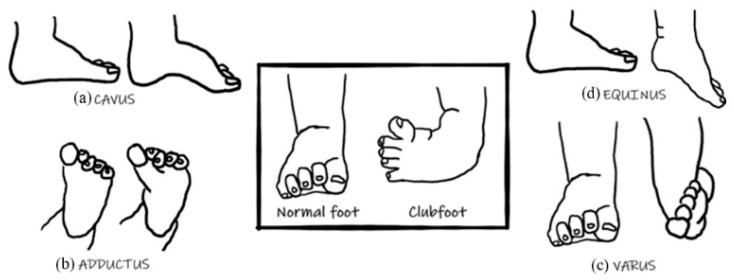
The four primary clubfoot abnormalities (CAVE).

Approximately 1–2 out of 1000 babies are born with talipes equinovarus, commonly referred to as clubfoot [1]. This condition is characterized by the foot’s inward and downward deformation, as depicted in Figure 1. In most cases, this deformity is not linked to other congenital issues, and approximately half of the cases involve both feet (bilateral) [2]. If left untreated, clubfoot can hinder mobility and become painful as the child ages. Clubfoot comprises four primary deformities: Cavus, Adductus, Varus, and Equinus (CAVE) [1,3,4], as shown in Figure 1. Cavus: The foot’s arch is higher than usual because the first metatarsal is plantarflexed about the calcaneum and hindfoot (Figure 1a). Adductus: The forefoot is adducted towards the midline. The navicular moves medially and starts to dislocate off the talus. The calcaneum also rotates medially under the talus as part of the Adductus deformity (Figure 1b). Varus: movement towards the midline. The heel is in varus about the tibia (Figure 1c). Equinus: an increase in the plantar flexion of the foot. The entire foot points downward towards the tibia (Figure 1d) [5]. The deformity correction involves Derotation around the talus (center of rotation of the deformity) of the calcaneal-forefoot block [6]. Therefore, Derotation is also used as one of the parameters to quantify the deformity.

**Figure 2:**
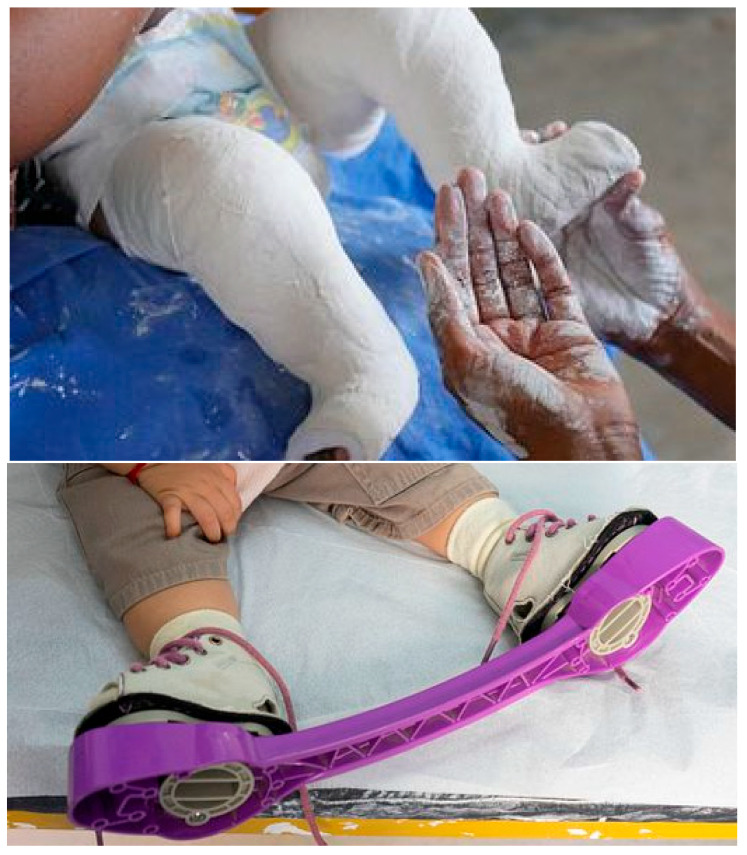
Ponseti Correction Method: Casting (**top**) and Bracing (**bottom**) [10].

The gold standard of clubfoot treatment is based on the non-surgical Ponseti method developed by Dr. Ignacio Ponseti in the 1960s. In this method, a patient’s clubfoot is subjected to serial long-leg plaster casting, as shown in Figure 2 (top), to manipulate the foot and slowly bring it back to its correct position. The casting process typically spans 5 to 7 weeks, during which a pediatric orthopedic surgeon performs weekly manipulations of the foot following the Ponseti method. At the same time, a specialist, such as a nurse practitioner, applies the cast.

The cast is cut and replaced each week by the clinicians. For 90% of cases, a heel cord tenotomy (cutting the tendon) is required to fully correct Equinus before applying the final cast [7]. After successive multi-stage correction, the foot is put into a specialized brace and bar system for approximately 23 h daily for 3–4 months, and then about 8–12 h a day (night-time) until 4 years of age [1,8,9] as shown in Figure 2 (bottom).

Although Ponseti treatment success has been demonstrated at older ages [11,12], beginning treatment as early as possible is often recommended. In infants, the Ponseti method typically does not involve imaging modalities such as CT or MRI unless surgical intervention is necessary. These imaging techniques are infrequently used and are generally not recommended due to the requirement for general anesthesia to immobilize the foot during the procedure. As a result, no information is available on the internal biomechanics of the clubfoot. Therefore, we propose to use surface information for finite element analysis (FEA) to simulate and study the successive clubfoot correction.

FEA has revolutionized the field of biomechanics by providing a powerful computational tool to simulate and analyze the complex behaviors of biological tissues and systems under various physiological conditions. This technique involves discretizing a complex structure into smaller, manageable elements, allowing for the detailed study of the stress, strain, and deformation of biological materials. FEA’s application in biomechanics spans a broad spectrum, from understanding the mechanical properties of bones and soft tissues [13,14] to designing medical implants [15,16] and prosthetics [17,18]. By creating detailed models of anatomical structures, FEA enables researchers and clinicians to predict how these structures respond to different forces, which is crucial for developing effective treatments and interventions. For example, in orthopedics, FEA helps design and optimize implants by simulating their interaction with the surrounding bone tissue, improving their performance and longevity. Advanced FEA models incorporate these properties to provide more accurate and reliable predictions, thereby enhancing our understanding of tissue mechanics and aiding in the development of bioengineered solutions. Several studies have employed FEA to investigate clubfoot but have not explicitly assessed the Ponseti method. For example, a study used FEA to evaluate midfoot von Mises stress levels in 17 participants suffering from clubfoot [19] with an average age of 25. Another study evaluated the optimal hindfoot correction angle while correcting the hindfoot deformity in patients with stiff clubfoot [20]. A CT-generated model of a 26-year-old male patient was used for the same study. Also, one study talked about personalized treatment and correction, but was based on a CT scan model of a 3-year-old [21].

In this study, we propose an iterative Finite Element Analysis (iFEA) method that utilizes a simplified, homogeneous clubfoot model to generate corrected clubfoot models, closely approximating the outer surface shape of a realistic clubfoot correction. This approach enables the use of 3D surface models of the clubfoot obtained from a 3D scanner, eliminating the need for volumetric imaging techniques such as MRI or CT scans. This paper simulates the clubfoot treatment to output intermediate corrected clubfoot models using homogeneous material using the finite element technique. These intermediate corrected clubfoot models could generate 3D printable casts similar to the plaster cast that surgeons generate during the conventional Ponseti treatment.

Ultimately, this proof-of-concept study aims to demonstrate the feasibility of reproducing surface-level geometric corrections consistent with the Ponseti trajectory, rather than replicating the exact clinical applications.

## 2. Methods

The treatment for clubfoot rarely involves MRI/CT scans; therefore, we are proposing a method that only uses surface information to simulate the Ponseti correction. Considering that assumption, we model a sample clubfoot model as a homogeneous, hyper-elastic model with properties like soft tissue. Manual Iterative FEA permanently reshapes a homogeneous surface model by exerting forces/torques with specific boundary conditions in multiple iterations. We also validate this surface model as a viable model to create corrected intermediate clubfoot models required to generate 3D printable casts.

The following subsections explain the step-by-step process from preprocessing the clubfoot model to correcting the deformity using the proposed iterative FEA.

### 2.1. Generating a 3D Surface Model

**Figure 3:**
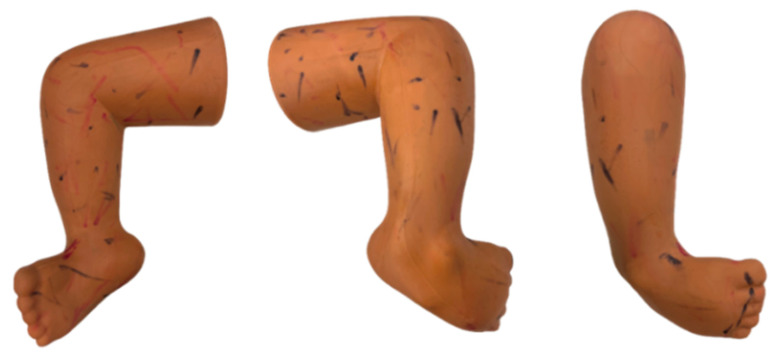
Original Rubber Clubfoot Model (3 different views).

An off-the-shelf training clubfoot rubber model (right leg) [MD Orthopaedics, Inc., Wayland, IA, USA] (Figure 3) was used for this FEA study. First, a 3D digital model of this clubfoot model was created by scanning this training model.

**Figure 4:**
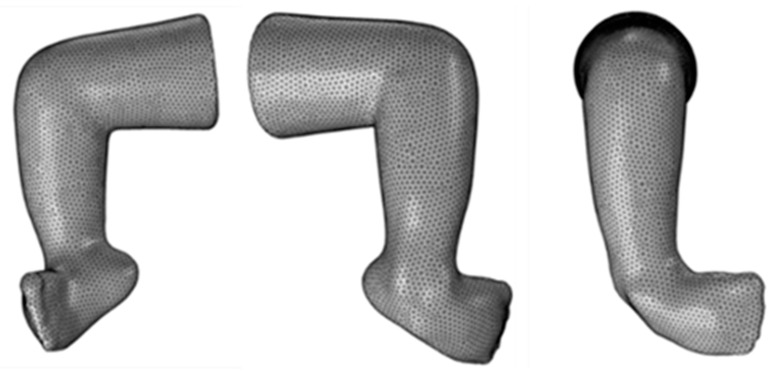
3D-model created using FARO Design Scan Arm and preprocessed using Meshmixer.

The handheld FARO^®^ Design Scan Arm (FARO Technologies, Inc, Lake Mary, FL, USA) was used for scanning with a system accuracy of 100 µm^2^ and probing accuracy of 75 µm [22]. Before exporting the model to the FEA software, the model was preprocessed to remove irregularities in the mesh. Then, an open-source meshing software, Meshmixer [Autodesk, Inc., San Rafael, CA, USA], was used to shrink-wrap the model, removing rough surfaces and edges, and creating a uniform mesh of elements 1 mm in size. After preprocessing, the model (Figure 4) was exported to the COMSOL Multiphysics (version 5.5) platform [COMSOL, Inc., Stockholm, Sweden] for Finite Element Analysis.

### 2.2. Landmark Selection and Initial Angle Calculation

**Figure 5:**
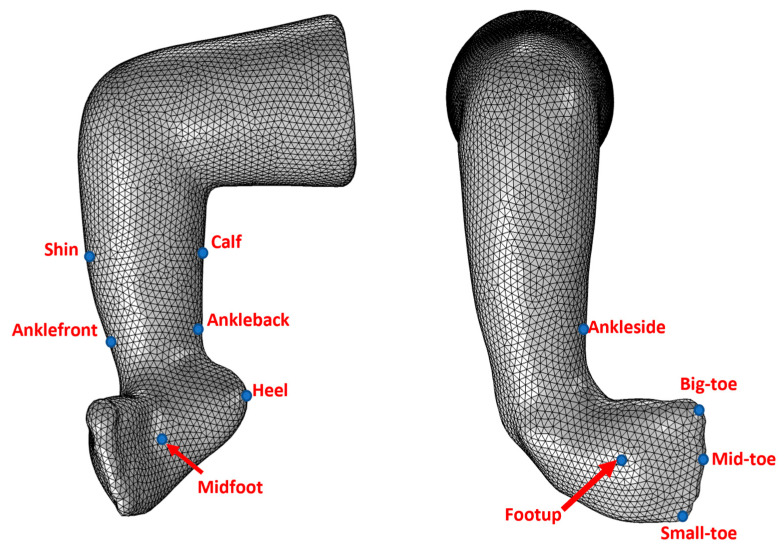
Anatomical landmarks on the 3D model [23].

In the COMSOL workspace, the imported hollow stereolithography model was converted to a solid body. An automatic meshing tool generated a mesh surface with elements of size 1 mm, which resulted in 52,179 nodes and 29,715 elements. Anatomical landmarks (like ankle, big-toe, mid-toe, small-toe, etc.) were defined using the domain point probe by manually selecting the point location. Figure 5 shows the landmarks we use to estimate the clubfoot deformity angles.

**Figure 6:**
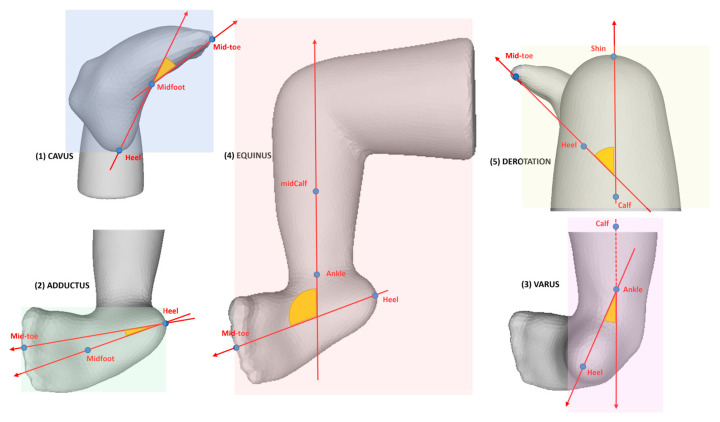
Reference angles at the initial stage: (**1**) Cavus, (**2**) Adductus, (**3**) Varus, (**4**) Equinus, (**5**) Derotation [23].

Three landmarks are required for each angle to define the different angles and quantify each deformity angle. Using Equation (1), vectors $\vec{A}$ and $\vec{B}$ are estimated from two pairs of landmarks for the corresponding deformity angle.

(1) $$\vec{PQ}=(x_{q}-x_{p},y_{q}-y_{p},z_{q}-z_{p})$$

where $\vec{PQ}$ is a vector connected by two points, *P* and *Q*, in a 3D space with corresponding *x*, *y*, and *z* coordinates. Then, each reference angle, i.e., Cavus, Adductus, Varus, Equinus, and Derotation, was calculated using vector dynamics using Equation (2) and corresponding vectors, as shown in Figure 6. For each vector corresponding to each deformity angle, we use different pairs of landmarks, as shown in our previous paper [23].

Table 1 shows the deformity angle, initial deformity, and desired deformity angles for a fully corrected clubfoot rubber model. Angles were calculated using simple vector dynamics, as shown in Equation (2).

(2) $$\cos\theta =\frac{\vec{A}\cdot \vec{B}}{\left|A\right|\left|B\right|}$$

where *θ* is the angle between two vectors $\vec{A}$ and $\vec{B}$.

The desired derotation angle is negative as, at the end of the Ponseti Method, the foot is constantly manipulated to point outwards and tends to return. These estimated initial angles could then be used to determine the severity of the deformity [6] and quantified using DiMeglio and Pirani scores. DiMeglio and Pirani scoring systems are used widely to quantify clubfoot deformity [24]. The desired angles will determine how much each angle should be adjusted from its initial value to achieve the corrected clubfoot position.

### 2.3. Material Model and FEA Simulation

Since the focus of this study is to reshape the surface model of the foot independent of the material, the exact biomechanical properties of the foot were not considered; the model was assumed to be homogeneous and was assigned material properties of soft tissue, which is a hyper-elastic material. The Mooney-Rivlin 5-parameter model, one of the hyper-elastic material models in COMSOL, was used to define the material parameters derived from [25], listed in Table 2. The strain energy equation for the material model is as follows:

(3) $$W_{s}\;=\;C_{10}\;(I_{1}\;-\;3)\;+\;C_{01}\;(I_{2}\;-\;3)\;+\;C_{20}\;(I_{1}\;-\;3{)}^{2}\;+\;C_{02}\;(I_{2}\;-\;3{)}^{2}\;+\;C_{11}\;(I_{1}\;-\;3)\;(I_{2}\;-\;3)\;+\;\frac{1}{2}\kappa \;{\left(J_{\mathrm{el}}\;-\;1\right)}^{2}$$

where W_s_ is the strain energy function, C_ij_ and κ (bulk modulus) are the material constants, and J_el_ is an invariant representing the ratio of the deformed elastic volume to the reference (undeformed) volume of materials. These material constants were inserted in the material definition in the COMSOL workspace. The value of the bulk modulus (κ) is derived from:

(4) $$\kappa \;=\;\frac{2}{D_{1}}$$

After preprocessing the model, boundary conditions were applied. The zero-displacement condition was applied to the upper ankle/tibia region to mimic the leg being held stationary during casting. Two different boundary conditions were used to manipulate the part of the model that needed to be corrected: a boundary condition to apply a 3D force with x, y, and z components, and a boundary condition to hold the upper foot while applying a 3D torque/moment to rotate the affected part of the model. These conditions are discussed in more detail in the following sections.

After defining the boundary conditions, the setup was simulated using a time-dependent study in the solid mechanics module of COMSOL. The time-dependent study was chosen to apply a gradual load. The simulation only converges if the load is applied gradually due to the hyper-elastic properties of the material. Also, applying loads gradually and iteratively saves the results for each step, which can be used to extract intermediate correction stages. The following section explains the manual iFEA process we implemented to reach the desired angles.

### 2.4. Iterative FEA Process Implementation

**Figure 7:**
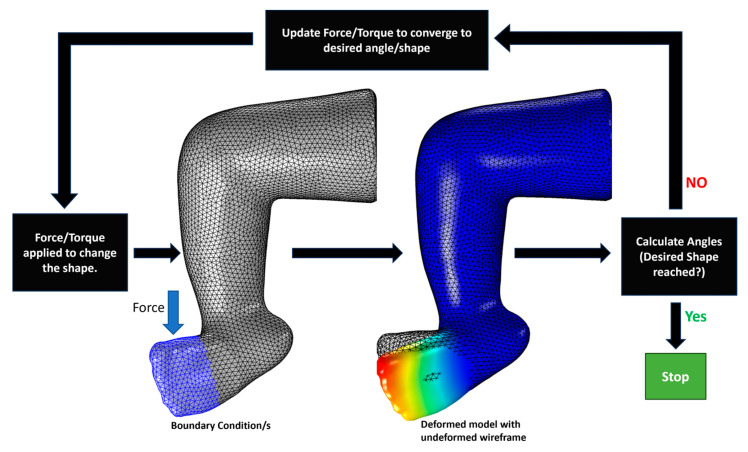
Manual Iterative FEA.

Figure 7 shows our proposed iFEA approach. In this paper, we implemented the iFEA technique manually, requiring us to repeat each iteration manually. This involved manually adjusting the direction and magnitude of the forces based on visual feedback while closely monitoring the clubfoot deformity angles (Cavus, Adductus, Varus, Equinus, and Derotation) during each iteration, informing us about the amount of deviation from the desired angles, which allowed us to adjust the force and torques accordingly. The applied forces and torques served only as computational drivers to achieve the target geometry and are not intended to represent clinician-applied magnitudes. This process was repeated until the desired corrected clubfoot shape was generated.

The following steps were used to correct the deformity, like the Ponseti method using manual iterative FEA (The figures shown in this section depict one of the intermediate iterations of a corrective step):

#### 2.4.1. Correcting Adductus and Cavus

**Figure 8:**
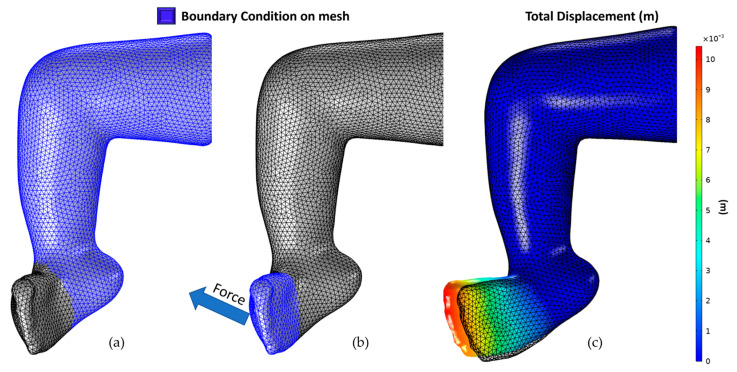
Cavus correction: (**a**) fixed boundary condition, (**b**) force boundary condition, and direction, (**c**) simulation result (displacement).

Figure 8 shows the boundary conditions applied. The boundary conditions represent how the specialist would fix a patient’s ankle/leg during casting. First, a force of magnitude 9.8 Newtons was applied in the direction shown in Figure 8 to move the part of the foot to the correct position, making the Cavus angle closer to zero. The magnitude and direction of this force were chosen after the initial investigation of the 3D model behavior when different random forces were applied.

**Figure 9:**
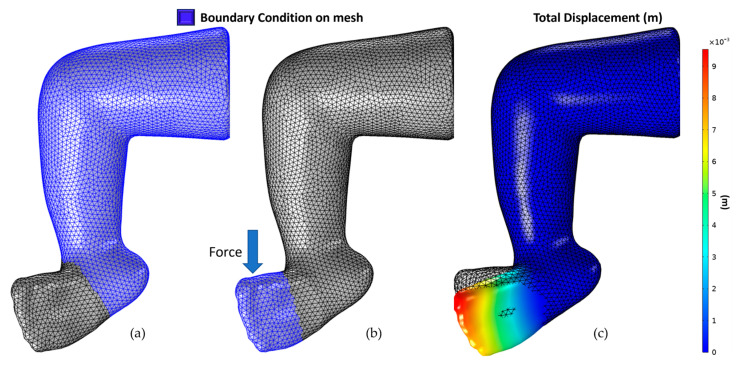
Adductus correction: (**a**) fixed boundary condition, (**b**) force boundary condition, and direction, (**c**) simulation result (displacement).

Similarly, a force of magnitude 5.1 Newtons was applied in the direction (parallel to the shin), as shown in Figure 9, to correct the Adductus and bring the angle closer to zero.

#### 2.4.2. Correcting Varus

**Figure 10:**
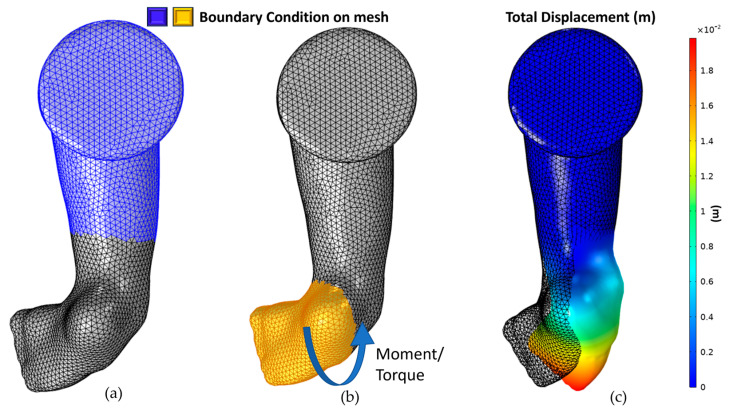
Varus correction: (**a**) fixed boundary condition, (**b**) moment boundary condition, and direction, (**c**) simulation result.

After correcting the Cavus and Adductus, the foot was rotated at the ankle to correct the Varus. The boundary conditions are shown in Figure 10. A moment or torque of 4.7 Newton-meters was applied using the rigid connector boundary condition.

#### 2.4.3. Correcting Equinus and Derotation

**Figure 11:**
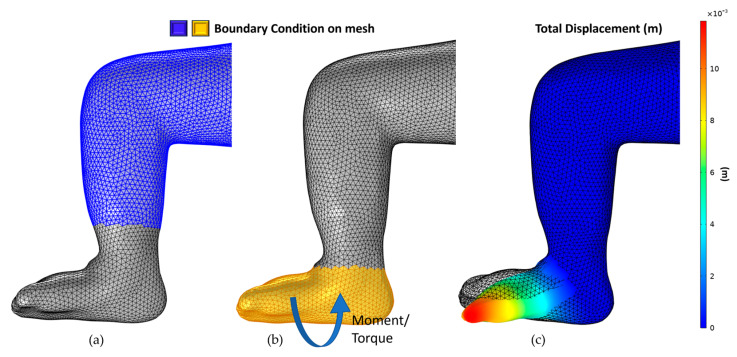
Equinus correction: (**a**) fixed boundary correction, (**b**) moment boundary condition, and direction, (**c**) simulation result.

**Figure 12:**
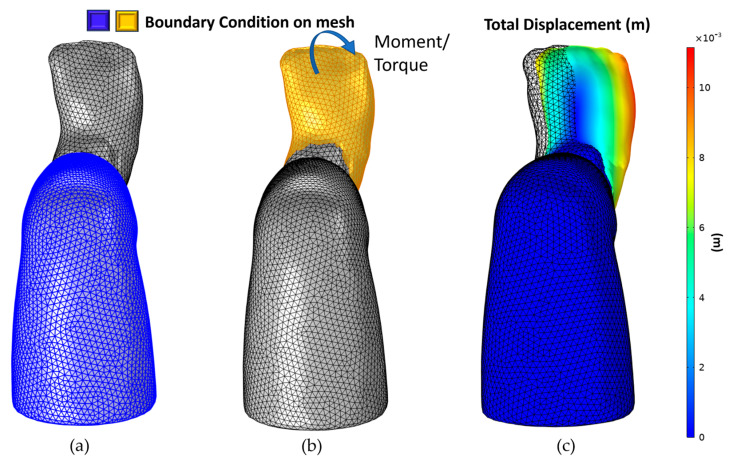
Derotation correction: (**a**) fixed boundary correction, (**b**) moment boundary condition, and direction, (**c**) simulation result.

After Varus’s correction, Equinus was corrected using the same boundary condition to reach 90 degrees by applying a moment or torque of 3.6 Newton meters, as shown in Figure 11. Like Equinus, Derotation was corrected by applying a moment or torque of 12.3 Newton meters, as shown in Figure 12.

### 2.5. Justifying and Validating the Use of a Homogeneous Model

**Figure 13:**
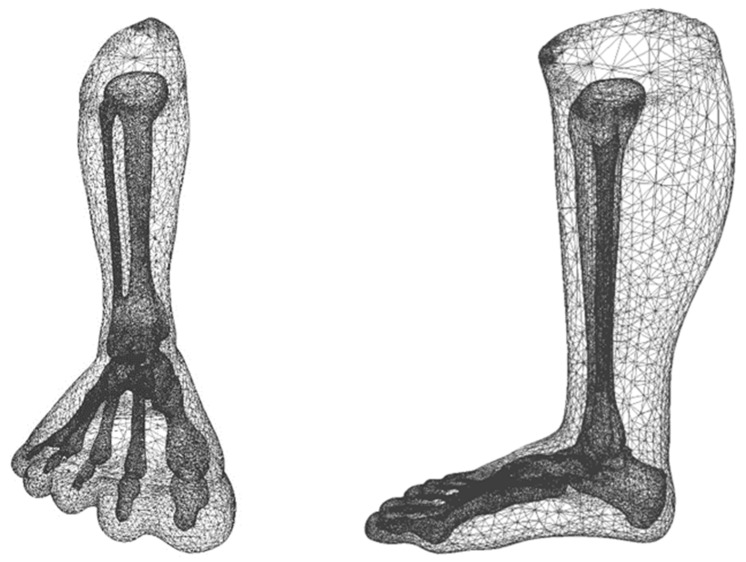
Adult Leg Model with Bones.

We wanted to compare the use of homogeneous and non-homogeneous (more biofidelic) models. An adult leg model comprising bone structure (Figure 13) was taken for the study from an open CAD library [26], using which the two models were defined: one having the properties of just the soft tissue and the other having the properties of both soft tissue and bone.

**Figure 14:**
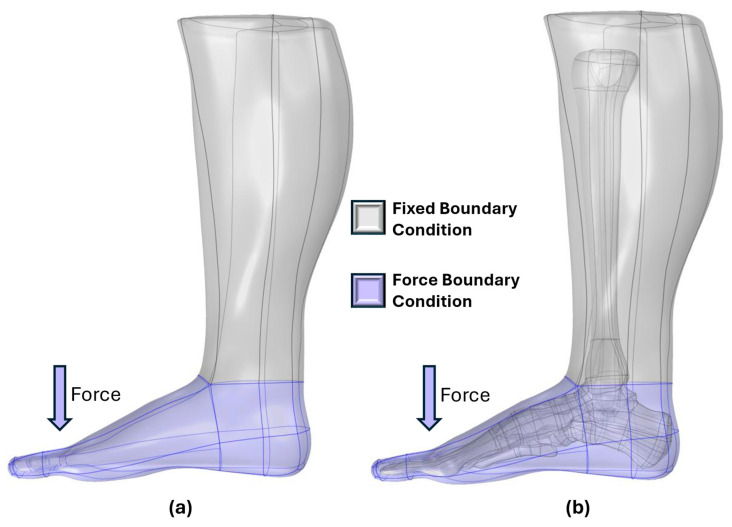
Foot model without bony structure (**a**), Foot model with bony structure (**b**).

Considering that the foot has a fully developed bony structure, this represents an extreme case; we know the bones are not fully developed in infants. The generated models and their boundary conditions (fixed and force) for these models are shown in Figure 14.

Finite element analysis was performed on these models to achieve the same deformation using varying force boundary conditions.

## 3. Results

### 3.1. Ponseti Simulation Study on a Rubber Model

**Figure 15:**
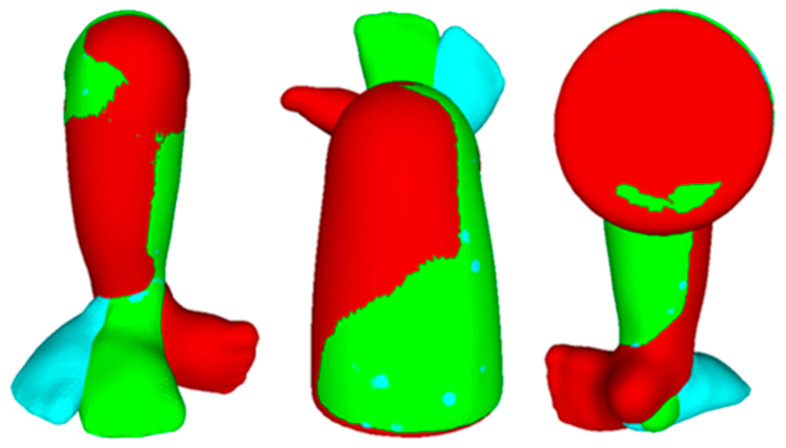
Correction results (Initial Model: Red, Fully Corrected: Green, Over-corrected: Blue).

Using Finite Element Analysis with COMSOL simulations, the clubfoot deformity in the training model was successfully corrected in six steps (Figure 15). The desired correction angles in each step were chosen, from intermediate iterations, according to the Ponseti method from the literature. The resulting angles had an average error of ±3.24°, as shown in Table 3.

In the first step, Cavus and Adductus were reduced significantly from approximately 30° and 15° to 2° and 3°, respectively. Due to this deformation, there were some changes in other angles, which is expected due to changes in the position of the landmarks. In the next few steps, Varus, Equinus, and Derotation were corrected, and the foot was fully corrected in step five. In the final step, the foot was over-corrected to obtain a Derotation angle of −40° (outwards). The angles were then measured to compare with the results of simulations.

The FEA model was assumed to be homogeneous and had the properties of soft tissue, as we were only interested in the foot’s geometry/outer surface. The magnitude and direction of the force used for manipulation were derived using the trial-and-error method to reach the desired angles. Completing the finite element analysis took about 2 h for each stage. We plan to automate this in the future using optimization algorithms, in which force will automatically and iteratively be calculated and applied to the surface model.

### 3.2. Results on Homogeneous and Non-Homogeneous Models

**Figure 16:**
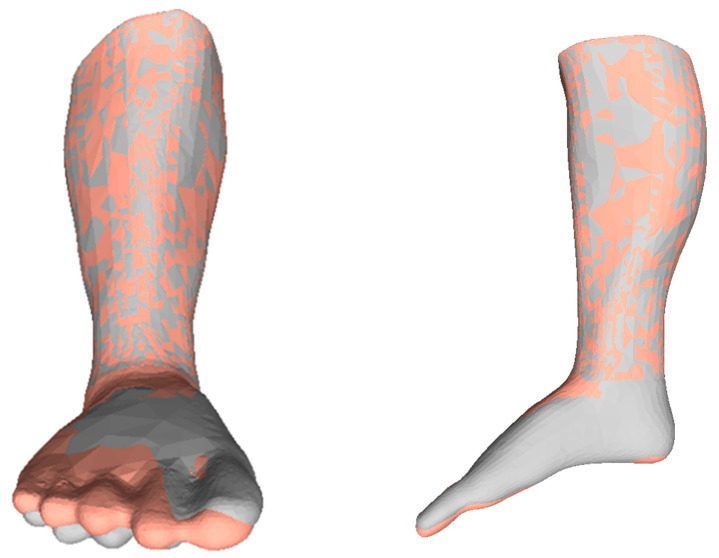
Comparing simulation results: complex model with soft tissue and bones (gray) and simple homogeneous model with properties of soft tissue (orange).

First, the non-homogeneous model with bones was manipulated using a force of up to 50 kN/m^2^, resulting in an Equinus angle of 115.12°. The time taken for the computation was 30 min (using a virtual computer provided by the University of Maryland). FEA was then performed on the homogeneous model to reach a similar deformation and Equinus angle; the force required for the same was 10 kN/m^2^, and the computation time was 10 min. The difference in the Equinus angle was 1.89°, and the average difference between points in the point cloud was 0.56 mm, as shown in Figure 16. The results suggest that using a simpler homogeneous model is reasonable, as it achieves the exact desired shape (and angles) and takes less time to compute.

## 4. Discussion

This study evaluated the feasibility of using iterative finite element analysis (iFEA) to simulate geometric corrections associated with the Ponseti method for clubfoot. Our results demonstrate that a simplified homogeneous foot model can be progressively reshaped into corrected configurations resembling those targeted during serial casting. Importantly, we found that the overall corrected surface geometry could be achieved regardless of whether the model incorporated heterogeneous structures such as bones, suggesting that computational efficiency can be gained with simpler models without sacrificing geometric outcomes.

However, it is essential to clarify the scope of our contribution. The applied forces and torques in this study (e.g., 9.8 N, 12.3 Nm) were not intended to replicate the exact magnitudes used by clinicians during Ponseti casting. Instead, they served as computational drivers to guide the iterative deformation of the model toward correction angles. In clinical practice, orthopedic surgeons apply subtle, low-magnitude forces guided by tactile feedback, which differ significantly from the values reported here. Thus, our approach should be interpreted as a proof-of-concept framework rather than a direct reproduction of the biomechanical loading conditions of infant feet. Future work will focus on quantifying clinician-applied forces during casting, for example, by embedding sensors into training models or casts, and incorporating these empirical data into the FEA framework.

Validation in this study was limited to geometric comparisons of simulated versus expected correction angles within the same modeling environment. While this provided an internal consistency check, it does not constitute independent biological validation. A critical next step will involve comparing the results against external reference standards, such as 3D scans or imaging data of patient feet before and after Ponseti correction, or controlled mechanical testing of physical foot models subjected to known loads. Such validation will ensure that the simulated corrections not only follow geometric targets but also align with clinically observed outcomes.

The iterative FEA approach presented here does not yet capture the complex, feedback-driven nature of the Ponseti method. Clinical casting involves subtle manipulations informed by tissue response and patient-specific variability, none of which were modeled in the present study. Instead, this work should be regarded as a conceptual demonstration that FEA can reproduce the overall geometric correction trajectory when driven by iterative loading conditions. Establishing this computational framework is a crucial first step toward developing more advanced, clinically realistic simulations.

Finally, the use of an adult/rubber model with homogeneous material properties represents a substantial simplification compared to the biomechanical reality of infant feet. Pediatric soft tissues, cartilage, and immature bones exhibit unique properties that were not captured here. While this limitation constrains the immediate translational value of our findings, it allowed us to isolate and evaluate the feasibility of the iFEA method. Ongoing and future work will focus on integrating pediatric-specific geometries (derived from surface scans or medical imaging) and tissue property data (obtained from the literature or cadaveric studies) to create models that more accurately reflect the clinical scenario.

Limitations: This study has several limitations that must be acknowledged:

- Force magnitudes: The applied forces and torques were arbitrary and computationally motivated, not measured from clinical practice. They should not be interpreted as physiologically realistic.
- Validation scope: The validation performed was internal and not based on independent patient or experimental data.
- Biological fidelity: The use of adult/rubber homogeneous models does not replicate the biomechanical properties of infant feet.
- Clinical inference: The study does not reproduce the tactile, feedback-based manipulation of the Ponseti method and therefore should not be interpreted as a full simulation of the clinical process.

Despite these limitations, this feasibility study establishes an iterative computational framework that can be refined in future work. By incorporating empirically measured forces, patient-specific geometries, and pediatric tissue properties, this approach has the potential to support patient-specific treatment planning, generate customized 3D-printable casts, and ultimately enhance the accessibility and precision of clubfoot correction.

## 5. Conclusions

This proof-of-concept study demonstrates that iterative finite element analysis (iFEA) can reproduce surface-level geometric changes consistent with the corrective trajectory of the Ponseti method, regardless of whether the model is homogeneous or contains internal bone structures. The work establishes a computational framework for exploring clubfoot correction but does not replicate the true biomechanical or feedback-driven process of clinical casting.

All applied forces and torques were computational drivers chosen to achieve the target geometry and should not be interpreted as physiological values. Validation was limited to internal geometric comparisons and requires extension to independent datasets, such as patient-specific imaging or controlled mechanical experiments.

Future development will focus on (1) measuring clinician-applied forces during Ponseti casting, (2) incorporating pediatric soft tissue properties and patient-specific geometries, and (3) performing external validation against clinical or experimental data. Achieving these steps will be critical for translating this computational approach into practical tools—such as patient-specific, 3D-printed casts—that could ultimately improve the precision, accessibility, and long-term outcomes of clubfoot treatment in children.

## Figures and Tables

**Table 1:** Clubfoot model initial angles.

Deformity/Angle	Initial Value (*°*)	Desired Value (*°*)
Cavus	35.55	0
Adductus	2.60	0
Varus	48.92	0
Equinus	109.18	90
Derotation	38.375	−40 (Outward)

**Table 2:** Material Parameters.

Material Constant	Value
C10	85,550 N/m^2^
C01	−58,400 N/m^2^
C20	38,920 N/m^2^
C02	−23,100 N/m^2^
C11	8484 N/m^2^
D1	4.370 × 10^−6^ m^2^/N

**Table 3:** Rubber Model Correction Angles in degrees.

Step	Target Deformity	Cavus (*°*)	Adductus (*°*)	Derotation (*°*)	Equinus (*°*)	Varus (*°*)
Initial		29.63	14.71	39.37	104.06	48.92
Step1	Cavus and Adductus	1.92	3.33	21.2	106.61	47.25
Step2	Varus	2.11	2.62	9.63	100.71	13.5
Step3	Varus, Equinus, and Derotation	3.56	2.59	4.48	90.89	11.97
Step4		6.24	5.11	2.88	92.86	1.56
Step5 (Fully Corrected)		7.64	5.52	9.74	93.39	1.32
Step6 (Over Corrected)	Derotation	8.01	6.35	−29.45	101.19	−2.22

## Data Availability

The data used to support the findings of this study are included in the article.
